# Impact of adjuvant chemotherapy and radiotherapy on tumour-infiltrating lymphocytes and PD-L1 expression in metastatic breast cancer

**DOI:** 10.1038/s41416-022-02072-2

**Published:** 2022-12-15

**Authors:** Shu Yazaki, Roberto Salgado, Tatsunori Shimoi, Masayuki Yoshida, Sho Shiino, Tomoya Kaneda, Yuki Kojima, Hitomi Sumiyoshi-Okuma, Tadaaki Nishikawa, Kazuki Sudo, Emi Noguchi, Takeshi Murata, Shin Takayama, Akihiko Suto, Yuichiro Ohe, Kan Yonemori

**Affiliations:** 1 Department of Medical Oncology, National Cancer Center Hospital, Tokyo, Japan; 2 Cancer Medicine, Jikei University Graduate School of Medicine, Tokyo, Japan; 3 Department of Pathology, GZA-ZNA Hospitals, Antwerp, Belgium; 4 Division of Research, Peter Mac Callum Cancer Centre, Melbourne, Australia; 5 Department of Diagnostic Pathology, National Cancer Center Hospital, Tokyo, Japan; 6 Department of Breast Surgery, National Cancer Center Hospital, Tokyo, Japan; 7 Department of Radiation Oncology, National Cancer Center Hospital, Tokyo, Japan; 8 Department of Thoracic Oncology, National Cancer Center Hospital, Tokyo, Japan

**Keywords:** Breast cancer, Cancer microenvironment

## Abstract

**Background:**

Chemotherapy and radiotherapy were postulated to induce an inflamed tumour microenvironment. We aimed to evaluate the effects of adjuvant chemotherapy/radiotherapy on tumour-infiltrating lymphocytes (TILs) and programmed death-ligand 1 (PD-L1) expression in metastatic breast cancer.

**Methods:**

We identified paired primary and metastatic tumours in 85 patients with breast cancer. Stromal TILs were assessed according to international guidelines. PD-L1 expression was evaluated using the VENTANA SP142 assay.

**Results:**

TILs were significantly lower in metastatic tumours than in primary tumours (12.2 vs. 8.3%, *p* = 0.049). PD-L1 positivity was similar between primary and metastatic tumours (21.2 vs. 14.1%, *p* = 0.23). TILs were significantly lower in patients who received adjuvant chemotherapy than in those who did not (−9.07 vs. 1.19%, *p* = 0.01). However, radiotherapy had no significant effect on TILs (*p* = 0.44). Decreased TILs predicted worse post-recurrence survival (hazard ratio, 2.94; 95% confidence interval [CI]: 1.41–6.13, *p* = 0.003), while increased TILs was associated with a better prognosis (HR, 0.12; 95% CI: 0.02–0.08, *p* = 0.04).

**Conclusions:**

TILs decreased in metastatic tumours, particularly in patients who relapsed after adjuvant chemotherapy. Changes in TILs from primary to metastatic sites could be a prognostic factor after recurrence.

## Introduction

Breast cancer is categorised into different subtypes according to hormone receptor (HR) and human epidermal growth factor receptor 2 (HER2) expression status. Triple-negative breast cancer (TNBC), characterised by the lack of HR and HER2 expression [1], most likely benefits from immune checkpoint inhibitors (ICIs) because tumour-infiltrating lymphocytes (TILs) and tumour mutation burden are higher in TNBC than in other subtypes [2, 3]. Recently, ICIs have been approved for use in metastatic TNBC and are being developed for the treatment of other subtypes [4, 5].

The immune-related tumour microenvironment (TME) affects the systemic treatment response and prognosis of cancer. TILs and programmed death-ligand 1 (PD-L1) expression can predict the prognosis and efficacy of ICIs in patients with breast cancer [6]. High TILs are associated with a favourable prognosis in early-stage breast cancer [2, 7–9] and with a higher efficacy of ICIs [10, 11]. PD-L1 expression is a companion diagnostic for ICIs used in metastatic TNBC, since their additive effect on chemotherapy is observed only in PD-L1-positive patients [12, 13].

The TME changes with tumour progression and cancer treatment [14]. In breast cancer, TILs and PD-L1 expression are lower in metastatic tumours than in primary tumours [15–17]. Chemotherapy and radiotherapy have immunomodulatory properties that make them ideal for use in combination with ICIs [18, 19]. The TONIC trial, which evaluated the immunomodulatory effects of 2-week induction treatments comprising chemotherapy or radiotherapy in patients with metastatic TNBC, suggested that chemotherapy and radiotherapy altered the TME and influenced ICI efficacy [20].

In localised breast cancer, adjuvant chemotherapy and radiotherapy can improve prognosis by eliminating micrometastases. However, their influence on metastatic TME remains unclear. It also remains unclear which tumour site (primary or metastatic) should be used for TILs/PD-L1-evaluation when ICI is considered in clinical practice, and whether post-treatment biopsies can be used for this purpose. Furthermore, the prognostic value of TILs/PD-L1 in metastatic tumours remains understudied. Therefore, we evaluated differences in TILs and PD-L1 expression in paired primary and metastatic tumours with or without adjuvant chemotherapy and radiotherapy. We also examined the prognostic role of TILs/PD-L1 in metastatic breast cancers.

## Methods

### Study population

We identified paired primary and metastatic samples from patients with breast cancer treated at the National Cancer Center Hospital (Tokyo, Japan). Eligible patients had the following criteria: breast cancer with recurrence, at least one tumour sample available from initial surgery between 2000 and 2018, and at least one tumour sample available from surgery or biopsy of relapsed disease. We excluded patients with insufficient tumour tissue available for assessing TILs and PD-L1 expression. Patients who received neoadjuvant chemotherapy were also excluded.

### Evaluation of TILs

TILs were assessed on haematoxylin/eosin-stained whole-tissue sections by two independent observers (SY and MY). TILs of the primary tumour were assessed in the surgical specimens. Stromal TILs were evaluated according to the International Immuno-Oncology Biomarker Working Group guidelines [21]. TILs were scored using semi-continuous (10% increment) methods.

### Immunohistochemical evaluation

Immunohistochemical staining for oestrogen receptor (ER), progesterone receptor (PR), and HER2 was performed using anti-ER (clone SP1; Roche Diagnostics K.K., Tokyo, Japan), anti-PR (clone 1E2; Roche Diagnostics K.K., Tokyo, Japan), and anti-HER2 antibodies (clone 4B5; Roche Diagnostics K.K., Tokyo, Japan), respectively. The HR status was considered positive if >10% of the tumour cells stained positive for ER or PR. HER2 expression was assessed via immunohistochemical analysis and scored according to the American Society of Clinical Oncology/College of American Pathologists guidelines. A positive status was defined as a score of 3+ on immunohistochemical analysis or HER2 amplification via fluorescent in situ hybridisation. PD-L1 expression was evaluated using the VENTANA SP142 assay (Roche Diagnostics K.K., Tokyo, Japan). PD-L1 positivity was defined according to the manufacturer’s recommendations. Results are reported as the percentage of PD-L1-stained tumour-infiltrating immune cells in the tumour area. A tumour was considered PD-L1 positive if ≥1% of tumour-infiltrating immune cells stained positive for PD-L1. PD-L1 positivity in ≥1% and <5% of tumour-infiltrating immune cells was reported as IC1, PD-L1 positivity in ≥5% and <10% as IC2, and PD-L1 positivity in ≥10% as IC3.

### Statistical analysis

Continuous numerical variables reported as median and mean were compared using non-parametric and parametric tests, respectively. Categorical variables were compared using the Chi-squared test. Stromal TILs ≥10% were considered high, consistent with previous studies [2, 11]. Changes in TILs between primary and metastases were classified as follows: an increase of ≥10% as increased TILs, a decrease of ≥10% as decreased TILs, and the remaining as showing no change. Changes in PD-L1 expression were classified as follows: an increase of ≥1 category in the IC score as increased PD-L1, a decrease of ≥1 category as decreased PD-L1, and the remaining as showing no change. Regarding the analysis of the association between radiotherapy and changes in TIL, we divided patients who received adjuvant radiotherapy into those with recurrence inside and those with recurrence outside the radiation field. Recurrence inside the field was defined as when a recurrence site had clearly developed in the radiation field. If the recurrence site partially overlapped the field edge and was difficult to classify, it was defined as recurrence outside the field. Logistic regression analysis was used to assess the relationship between changes in TILs, PD-L1 expression, and clinico-pathological factors. Post-recurrence survival was defined as the time from the first invasive recurrence (locoregional or distant) to any-cause death. The Kaplan–Meier method was used to estimate survival, and the results were compared using the log-rank test. We used a Cox proportional-hazards model to evaluate the prognostic value of clinicopathological factors. All tests were two-tailed, with the significance set at *p* < 0.05. Statistical analyses were performed using STATA (version 15.1; StataCorp, College Station, TX, USA) and GraphPad Prism, version 8.0 (GraphPad Software, San Diego, CA, USA).

## Results

### Patient characteristics

We identified 118 patients with paired primary and metastatic tumours. We excluded six patients with insufficient tumour samples and 27 patients who received neoadjuvant chemotherapy. Finally, 85 patients were included in this analysis. The characteristics of the patients are summarised in Table 1. Details regarding radiotherapy are available in the Supplemental Material.

**Table 1:** Patient characteristics.

	*N* = 85
Age at diagnosis, median (range)	55 (25–78)
≥65, *n* (%)	18 (21.2%)
Tumour size^a^, *n* (%)	
≥pT2	45 (52.9%)
Lymph node status^a^, *n* (%)	
1–3	21 (24.7%)
≥4	17 (20%)
Histology, *n* (%)	
Invasive ductal	80 (94.1)
Others	5 (5.9)
Histological grade^a^, *n* (%)	
1–2	33 (38.8%)
3	52 (61.2%)
Stromal TILs^a^	
Mean % (SD)	12.2 (17.3)
Median % (IQR)	10 (0–20)
PD-L1, *n* (%)	
IC1	16 (18.8)
IC2	1 (1.2)
IC3	1 (1.2)
Subtype^a^, *n* (%)	
HR-positive, HER2-negative	50 (58.8%)
HR-positive, HER2-positive	2 (2.4%)
HR-negative, HER2-positive	1 (1.2%)
Triple negative	32 (37.7%)
Breast surgery, *n* (%)	
Lumpectomy	48 (56.5%)
Mastectomy	37 (43.5%)
Axially lymph node dissection, *n* (%)	62 (72.9%)
Adjuvant radiotherapy, *n* (%)	46 (54.1%)
Whole breast	29 (34.1%)
Whole-breast and regional nodes	7 (8.2%)
Chest wall and regional nodes	10 (11.8%)
Adjuvant chemotherapy^b^, *n* (%)	43 (50.6%)
Chemotherapy regimen^c^, *n* (%)	
Anthracycline and taxane	20 (46.5%)
Anthracycline only	16 (37.2%)
Taxane only	3 (7.0%)
Others	4 (9.3%)
Hormone therapy, *n* (%)	37 (43.5%)
RFI, median (range)	54 (2–176) months
Visceral metastasis^d^, *n* (%)	17 (20%)
Biopsy sites of metastases, *n* (%)	
Breast	27 (31.8%)
Regional lymph nodes	23 (27.1%)
Chest wall	11 (12.9%)
Lung	10 (11.8%)
Liver	3 (3.5%)
Bone	3 (3.5%)
Brain	2 (2.4%)
Others^**e**^	6 (7.0%)

*HR* hormone receptor, *HER2* human epidermal growth factor receptor 2, *RFI* recurrence-free interval.

^a^For the primary tumour.

^b^Three patients received both neoadjuvant and adjuvant chemotherapy.

^c^*n* = 43.

^d^Visceral metastasis include liver, lung, brain metastasis.

^e^Cervical lymph nodes (*n* = 4), skin (*n* = 1), pancreas (*n* = 1).

### TILs and PD-L1 expression compared between primary and metastatic tumours

**Fig. 1:**
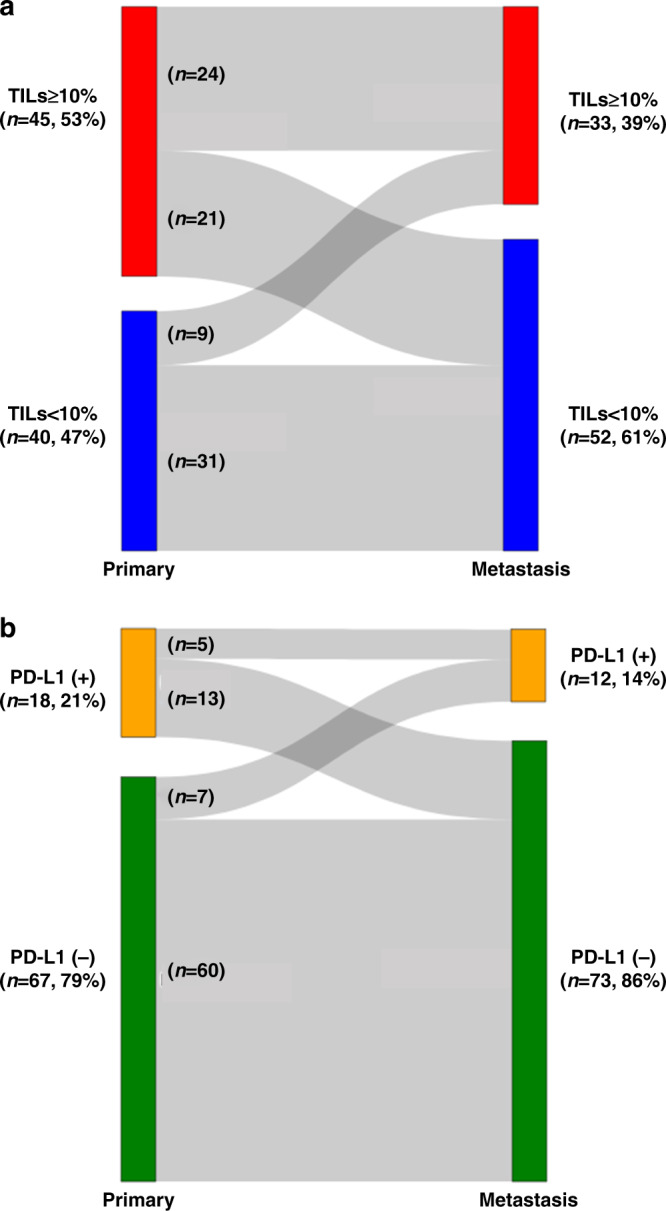
TILs and PD-L1 expression shifts between primary and metastatic tumours. **a** The proportion of patients with high stromal TILs in metastatic tumours (39%) is lower than that of those with high stromal TILs in primary tumours (53%). **b** The proportion of patients with PD-L1-positive metastatic tumours (14%) is also lower than that of those with PD-L1-positive primary tumours (21%). PD-L1 programmed death-ligand 1, TIL tumour-infiltrating lymphocyte.

The mean and median stromal TILs of primary tumours were 12.2% (standard deviation [SD], 17.3%) and 10% (interquartile range [IQR], 0–20.0%), respectively. The mean and median stromal TILs of metastatic tumours were 8.3% (SD, 14.3%) and 0% (IQR, 0–10%), respectively. The mean stromal TILs were significantly lower in metastatic tumours than in primary tumours (mean change [%], −4.0; 95% confidence interval [CI], −8.0 to −0.003; *p* = 0.049). The mean TIL change did not differ by metastasis site (loco-regional vs. distant, −5.25 vs. −1.15, *p* = 0.35), breast cancer subtype (TNBC vs. others, −5.63 vs. −3.01, *p* = 0.53) or recurrence-free interval (RFI) (≥median vs. <median, −4.10 vs. −3.90, *p* = 0.96). The proportion of high-TIL tumours was higher in primary tumours than in metastases (52.9 vs. 38.8%, *p* = 0.06). The proportion of high-TIL tumours in the breast, lymph node, and lung metastases was similar to that in primary tumours (high-TIL tumours: breast, 52.0%; lymph node, 48.0%; lung, 40.0%), whereas liver, bone, and brain metastases had no high-TIL tumours (Supplemental Fig. 1A). TILs decreased, increased, and remained unchanged in 33 (38.8%), 18 (21.2%), and 34 (40%) patients, respectively. TILs showed a discordance (high vs. low) between primary and metastatic tumours in 30 (35.3%) patients (high to low, *n* = 21; low to high, *n* = 9) (Fig. 1a).

PD-L1 positivity showed no differences between primary tumours and metastatic tumours (21.2 vs. 14.1%, *p* = 0.23), but varied according to the metastatic sites. Lung metastasis had the highest PD-L1 positivity rate (30%), whereas liver, bone, and brain metastases showed no PD-L1 positivity (Supplemental Fig. 1B). PD-L1 expression decreased, increased, and remained unchanged in 13 (15.3%), 9 (10.6%), and 63 (74.1%) patients, respectively. Discordance in PD-L1 expression (positive vs. Negative) between primary and metastatic tumours was observed in 20 (23.5%) patients (positive to negative, *n* = 13; negative to positive, *n* = 7) (Fig. 1b).

### Influence of adjuvant chemotherapy and radiotherapy on TILs and PD-L1 expression in metastatic tumours

**Fig. 2:**
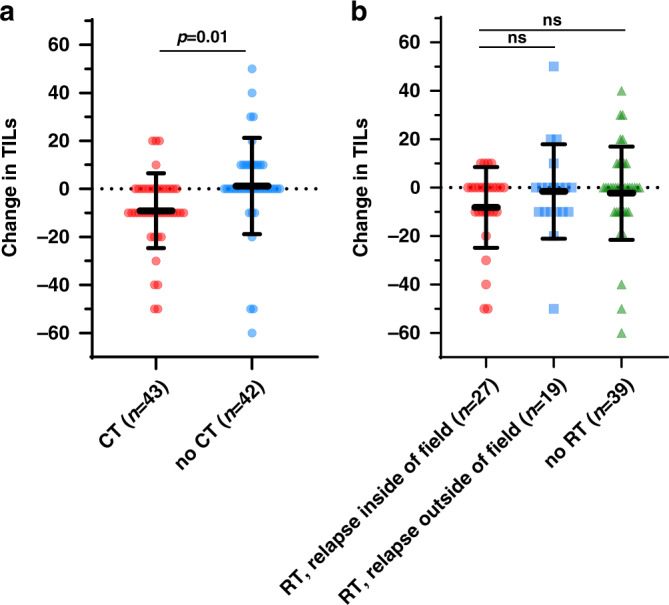
Changes in TILs with or without adjuvant chemotherapy and radiotherapy. Changes in TILs between primary tumours and metastases are compared in patients receiving or not receiving adjuvant chemotherapy (**a**) and radiotherapy (**b**). The radiotherapy group is divided into two subgroups: inside and outside of the field of recurrence. The bars represent the mean ± SD. CT chemotherapy, RT radiotherapy, SD standard deviation, TIL tumour-infiltrating lymphocyte.

The change in TILs in primary and metastatic tumours was significantly lower in patients receiving adjuvant chemotherapy than in patients not receiving it (mean change [%], −9.07 vs. 1.19, *p* = 0.01) (Fig. 2a). Decreased TILs were observed in 60.5% (26/43) of patients receiving adjuvant chemotherapy, and 16.7% (7/42) of patients not receiving adjuvant chemotherapy (*p* < 0.001). In the HR-positive HER2-negative group, there was no significant difference in mean TIL change with or without adjuvant chemotherapy (−6.67 vs. −1.72, *p* = 0.33). However, in the TNBC group, the mean TIL change was significantly lower in patients with adjuvant chemotherapy than those without (−11.36 vs. 7.0, *p* = 0.013) (Supplemental Fig. 2A, B).

The change in TILs in primary and metastatic tumours did not differ between patients who received adjuvant radiotherapy and those who did not (mean change [%], −5.44 vs. −2.31, *p* = 0.44). The change in TILs in primary tumours and metastatic tumours among those with recurrence inside the radiation field (*n* = 27), outside the radiation field (*n* = 19), and no previous radiotherapy (*n* = 39) was not significantly different (mean change [%], −8.15 vs. −1.58 vs. −2.31, *p* = 0.37) (Fig. 2b). Decreased TILs were observed in 44.4% (12/27), 42.1% (8/19), and 33.3% (13/39) of patients with recurrence inside the field, outside the field, and no previous adjuvant radiotherapy, respectively (*p* = 0.94). In the HR-positive HER2-negative group, there was no significant difference in mean TIL change between the three groups (inside vs. outside vs. no radiotherapy, −2.0 vs. 0 vs. −6.15, *p* = 0.6). However, in the TNBC group, the mean TIL change was significantly different between the three groups. The change in TILs was significantly lower in patients with recurrence inside the field than in those without adjuvant radiotherapy (−18.2 vs. −2.2 vs. 3.33, *p* = 0.024, Tukey’s multiple comparisons test *p* < 0.022 between inside and no radiotherapy) (Supplemental Fig. 2C, D). We further analysed patients who received adjuvant radiotherapy by dividing them into those with locoregional recurrence (*n* = 34) and those with distant metastasis (*n* = 12). There was no significant difference in the change in TILs (locoregional vs. distant vs. no radiotherapy, −6.77 vs. −1.67 vs. −2.31, *p* = 0.53) (Supplemental Fig. 3).

We performed a logistic regression analysis to determine the relationship between clinicopathological factors and decreased TILs. In the multivariable analysis, after adjusting the histological grade associated with decreased TILs in the univariable analysis, adjuvant chemotherapy remained significant (odds ratio, 6.85; 95% CI, 2.34–20.08; *p* < 0.001) (Table 2).

**Table 2:** Logistic regression analysis of decreased tumour-infiltrating lymphocytes.

	Univariate		*p* value	Multivariate		*p* value
	OR	95% CI		OR	95% CI	
Age (≥65 vs. <65)	0.74	0.25–2.21	0.59			
Tumour size (≥pT2 vs. <pT2)	2.04	0.83–4.99	0.12			
Nodal status (positive vs. negative)	2.36	0.97–5.75	0.06			
Subtype (TNBC vs. others)	1.39	0.57–3.41	0.47			
HG (3 vs. 1–2)	6.53	2.18–19.6	0.001	5.71	1.75–18.06	0.004
RFI (≥median vs. <median)	0.66	0.27–1.59	0.36			
Site of recurrence (distant vs. locoregional)	0.97	0.38–2.52	0.96			
Adjuvant CT (yes vs. no)	7.65	2.77–21.1	<0.001	6.85	2.34-20.08	<0.001
Adjuvant RT (yes vs. no)	1.54	0.64–3.73	0.34			
Adjuvant HT (yes vs. no)	1.39	0.58–3.35	0.46			

*CT* chemotherapy, *CI* confidence interval, *HG* histological grade, *HT* hormone therapy, *RT* radiotherapy, *RFI* recurrence-free interval, *OR* odds ratio, *RT* radiotherapy, *RFI* recurrence-free interval, *OR* odds ratio, *TNBC* triple-negative breast cancer, *TIL* tumour-infiltrating lymphocyte.

The rate of decreased PD-L1 expression with or without adjuvant chemotherapy or radiotherapy showed no significant difference. Decreased PD-L1 expression was observed in 18.6% (8/43) of patients receiving adjuvant chemotherapy and in 11.9% (5/42) of patients not receiving adjuvant chemotherapy (*p* = 0.39). Decreased PD-L1 expression was also observed in 18.5% (5/27), 10.5% (2/19), and 15.4% (6/39) of patients with recurrence inside the field, outside the field, and no previous adjuvant radiotherapy, respectively (*p* = 0.55).

### Impact of TILs and PD-L1 expression changes on post-recurrence survival

**Fig. 3:**
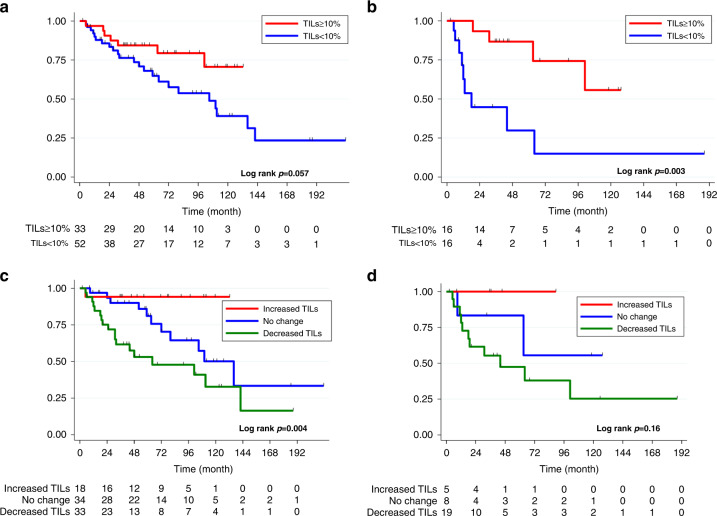
TILs and post-recurrence survival. Post-recurrence survival in patients with high TILs (≥10%) vs. low TILs (<10%) in metastatic tumours in the total population (**a**) and in the TNBC subgroup (**b**). Post-recurrence survival in patients according to the change in TILs (increased vs. no change vs. decreased) in the total population (**c**) and in the TNBC subgroup (**d**). TNBC triple-negative breast cancer, TIL tumour-infiltrating lymphocyte.

The median follow-up period was 52 months (IQR, 27–97 months), and 30 events were observed. High-TIL metastatic tumours tended to be associated with better post-recurrence survival (hazard ratio [HR], 0.45; 95% CI, 0.19–1.05; *p* = 0.057) in the total population, and was significantly associated with better post-recurrence survival in the TNBC group (HR, 0.19; 95% CI, 0.059–0.65; *p* = 0.003) (Fig. 3a, b). However, high-TIL primary tumours were not a favourable prognostic factor after recurrence both in the total population (HR, 1.77; 95% CI, 0.83–3.80; *p* = 0.14) and the TNBC group (HR, 1.75; 95% CI, 0.22–14.0; *p* = 0.59) (Supplemental Fig. 4A, B). The dynamic change in TILs between primary and metastatic tumours, all subtypes combined, significantly stratified post-recurrence survival (*p* = 0.004) (Fig. 3c). Patients with increased TILs were associated with a better prognosis (HR, 0.12; 95% CI 0.016–0.88; *p* = 0.037), and patients with decreased TILs were associated with worse prognosis (HR, 2.94; 95% CI, 1.41–6.13; *p* = 0.004). A similar trend was observed in patients with TNBC (*p* = 0.16) (Fig. 3d). PD-L1 positivity in primary or metastatic tumours was not associated with post-recurrence survival (PD-L1 positivity in primary tumours: HR, 0.92; 95% CI, 0.47–1.82; *p* = 0.81; PD-L1 positivity in metastases: HR, 0.23; 95% CI, 0.038–1.35; *p* = 0.10). The change in the PD-L1 IC score was also not associated with post-recurrence survival (*p* = 0.16). In the univariable analysis, decreased/increased TILs, larger tumour size, TNBC subtype, visceral metastasis (liver, lung, brain), and adjuvant chemotherapy were significantly associated with post-recurrence survival. The prognostic value of decreased or increased TIL was not confirmed in the multivariable analysis (Table 3).

**Table 3:** Cox proportional-hazards model for post-recurrence survival.

	Univariable		*p* value	Multivariable		*p* value
	HR	95% CI		HR	95% CI	
TILs in primary tumours (≥10 vs. <10%)	1.77	0.83–3.80	0.14			
TILs in metastatic tumours (≥10 vs. <10%)	0.45	0.19–1.05	0.065			
TIL changes						
No change	Ref	–	–	Ref	–	–
Decreased	2.94	1.41–6.13	0.004	1.36	0.50–3.66	0.55
Increased	0.12	0.016–0.88	0.0037	0.15	0.02–1.21	0.074
PD-L1 expression in primary tumours (positive vs. negative)	0.92	0.47–1.82	0.81			
PD-L1 expression in metastatic tumours (positive vs. negative)	0.23	0.038–1.35	0.1			
PD-L1 changes						
No change	Ref	–	–			
Decreased	1.85	0.79–4.33	0.16			
Increased	0.25	0.035–1.88	0.18			
Age^a^ (≥65 vs. <65)	1.54	0.72–3.31	0.27			
Tumour size^b^ (≥pT2 vs. <pT2)	2.31	1.06–5.06	0.036	1.49	0.61–3.67	0.38
Nodal status^b^ (positive vs. negative)	1.75	0.85–3.60	0.13			
Subtype^c^ (TNBC vs. others)	2.08	1.004–4.30	0.049	1.77	0.74–4.23	0.2
HG^b^ (3 vs. 1–2)	1.99	0.91–4.39	0.087			
RFI (≥median vs. <median)	0.86	0.41–1.80	0.69			
Visceral metastasis^d^ (yes vs. no)	2.52	1.08–5.87	0.032	2.76	1.07–7.11	0.04
Adjuvant CT (yes vs. no)	4.26	1.90–9.54	<0.001	2.23	0.87–5.70	0.095
Adjuvant RT (yes vs. no)	0.97	0.47–2.01	0.95			
Adjuvant HT (yes vs. no)	0.83	0.40–1.73	0.62			

^a^At recurrence.

^b^For primary tumours.

^c^For metastatic tumours.

^d^Visceral metastasis include liver, lung, brain metastasis.

*CT* chemotherapy, *CI* confidence interval, *HR* hazard ratio, *HG* histological grade, *HT* hormone therapy, *RT* radiotherapy, *RFI* recurrence-free interval, *PD-L1* programmed death-ligand 1, *TNBC* triple-negative breast cancer, *TIL* stromal tumour-infiltrating lymphocyte.

## Discussion

To the best of our knowledge, this is the first study to evaluate changes in TILs and PD-L1 expression, and their association with adjuvant chemotherapy/radiotherapy, using paired samples from primary tumours and metastatic tumours.

We demonstrated that TILs were significantly lower in metastatic tumours than in primary tumours, especially in patients receiving adjuvant chemotherapy. In the TONIC study, TILs and immune-related signatures increased after short-term doxorubicin treatment, which was inconsistent with our results [20], although immune-induction after chemotherapy may be dependent on the chemotherapy drugs used. Park et al. [22] reported that TILs significantly increased after one cycle of neoadjuvant doxorubicin/cyclophosphamide treatment in patients with pathological complete response, but not in those with residual disease. Several studies have also reported that TILs decreased in patients with residual disease after neoadjuvant chemotherapy [23–27]. In the advanced setting, a post hoc analysis of the KEYNOTE-086 trial also confirmed that TILs were also lower in previously treated metastatic TNBCs than in previously untreated ones (5 vs. 17.5%, *p* < 0.001) [10]. These studies suggest that, although short-term chemotherapy may increase TILs, chemo-resistant tumours may have lower TILs because of immune evasion. Our results suggest that immune-evasive phenotypes of cancer cells not eradicated with adjuvant chemotherapy may have been selected and evolved in recurrent tumours. The KEYNOTE-355 and IMpassion130 trials evaluated the efficacy of pembrolizumab or atezolizumab when added to standard chemotherapy for untreated metastatic TNBC [12, 13, 28]. These trials included patients who relapsed after receiving adjuvant chemotherapy. Although the benefits of ICIs on progression-free survival or overall survival were generally consistent across subgroups, the point estimate of HR was closer to 1.00 in patients who received adjuvant chemotherapy than in those who did not. The smaller benefit of ICIs in patients receiving adjuvant chemotherapy might be due to the decreased TILs in metastatic tumours.

In clinical practice, the question “where should we take a biopsy?” is often asked. Thus far, no clear answer can be provided. Nearly half of patients in the IMpassion130 trial had PD-L1 assessed in the primary tumour, and PD-L1 positivity in either primary or metastatic tumour was associated with improved survival with atezolizumab [11]. Our data suggest that, considering the dynamic nature of the immune system, it is better to biopsy a site where the highest number of TILs can be expected, namely in the primary tumour, lymph nodes, and lung. Furthermore, taking a biopsy in the untreated setting is always preferable, since TILs decrease after adjuvant chemotherapy. Hence, our results may help answer a pertinent and still unanswered question relevant to routine practice.

Changes in TILs and PD-L1 expression were similar between patients who received adjuvant radiotherapy and those who did not, regardless of the recurrence site. Previous pre-clinical studies demonstrated that radiotherapy increased T-cell infiltration and enhanced the efficacy of ICIs [29, 30]. Studies comparing pre- and post-chemoradiotherapy tumour samples in patients with NSCLC also showed that TILs increased after chemoradiotherapy [31, 32]. However, our results were different, possibly because we evaluated post-recurrence tumour samples that were not collected immediately after treatment.

We also evaluated whether the changes in TILs and PD-L1 expression affected post-recurrence survival. Several retrospective studies evaluating the prognostic role of TILs in metastatic tumours for recurrent breast cancer have reported inconsistent results [15, 16, 33, 34]. In our study, high metastatic TILs were associated with favourable post-recurrence survival in patients with TNBC, while high primary TILs were not. Moreover, the dynamic changes in TILs between primary and metastatic tumours were predictive of post-recurrence survival, and decreased TILs in metastatic tumours were associated with a poor prognosis. Our results suggest that TILs also provide prognostic information in the metastatic setting. Further development of immune-induction strategies to increase TILs in metastases may improve the prognosis of recurrent breast cancer.

This study is limited by its retrospective design and small sample size. However, given that biopsies or diagnostic surgery of metastases are often omitted, the value of our study lies in its inclusion of a large patient cohort based on a unique data source, which allowed us to evaluate shifts in the TME in the same patients between primary tumours and metastatic tumours. In the future, further validation in multi-centre studies with a larger number of cases is desirable. Furthermore, patients with various breast cancer subtypes were included. TILs and PD-L1 expression, and their prognostic significance varied by subtype. Extrapolation of our findings to HER2-positive breast cancer should be performed with caution, since HER2-positive cases accounted for only 3.6% of our study sample.

In conclusion, TILs were decreased in metastatic tumours, particularly after adjuvant chemotherapy, resulting in a poor prognosis. In contrast, increased TILs were associated with a better prognosis in the total patient sample, and high TILs in metastatic tumours were associated with a better prognosis in patients with TNBC. The evaluation of TILs in metastatic tumours may help predict the prognosis and aid the development of personalised immunotherapy.

## Supplementary information

All authors agreed with the final manuscript

Supplemental material

## Data Availability

The data analysed in this study are available from the corresponding author upon reasonable request.

## References

[1] Parise CA, Bauer KR, Brown MM, Caggiano V (2009). Breast cancer subtypes as defined by the estrogen receptor (ER), progesterone receptor (PR), and the human epidermal growth factor receptor 2 (HER2) among women with invasive breast cancer in California, 1999–2004. Breast J.

[2] Denkert C, von Minckwitz G, Darb-Esfahani S, Lederer B, Heppner BI, Weber KE (2018). Tumour-infiltrating lymphocytes and prognosis in different subtypes of breast cancer: a pooled analysis of 3771 patients treated with neoadjuvant therapy. Lancet Oncol.

[3] Budczies J, Bockmayr M, Denkert C, Klauschen F, Lennerz JK, Györffy B (2015). Classical pathology and mutational load of breast cancer – integration of two worlds. J Pathol Clin Res.

[4] Adams S, Gatti-Mays ME, Kalinsky K, Korde LA, Sharon E, Amiri-Kordestani L (2019). Current landscape of immunotherapy in breast cancer: a review. JAMA Oncol.

[5] Noguchi E, Shien T, Iwata H (2021). Current status of PD-1/PD-L1 blockade immunotherapy in breast cancer. Jpn J Clin Oncol.

[6] Gonzalez-Ericsson PI, Stovgaard ES, Sua LF, Reisenbichler E, Kos Z, Carter JM (2020). The path to a better biomarker: application of a risk management framework for the implementation of PD-L1 and TILs as immuno-oncology biomarkers in breast cancer clinical trials and daily practice. J Pathol.

[7] Savas P, Salgado R, Denkert C, Sotiriou C, Darcy PK, Smyth MJ (2016). Clinical relevance of host immunity in breast cancer: from TILs to the clinic. Nat Rev Clin Oncol.

[8] Park JH, Jonas SF, Bataillon G, Criscitiello C, Salgado R, Loi S (2019). Prognostic value of tumour-infiltrating lymphocytes in patients with early-stage triple-negative breast cancers (TNBC) who did not receive adjuvant chemotherapy. Ann Oncol.

[9] Loi S, Drubay D, Adams S, Pruneri G, Francis PA, Lacroix-Triki M (2019). Tumour-infiltrating lymphocytes and prognosis: a pooled individual patient analysis of early-stage triple-negative breast cancers. J Clin Oncol.

[10] Loi S, Adams S, Schmid P, Cortés J, Cescon DW, Winer EP (2017). Relationship between tumour infiltrating lymphocyte (TIL) levels and response to pembrolizumab (pembro) in metastatic triple-negative breast cancer (mTNBC): results from KEYNOTE-086. Ann Oncol.

[11] Emens LA, Molinero L, Loi S, Rugo HS, Schneeweiss A, Diéras V (2021). Atezolizumab and nab-paclitaxel in advanced triple-negative breast cancer: biomarker evaluation of the IMpassion130 study. J Natl Cancer Inst.

[12] Schmid P, Adams S, Rugo HS, Schneeweiss A, Barrios CH, Iwata H (2018). Atezolizumab and nab-paclitaxel in advanced triple-negative breast cancer. N Engl J Med.

[13] Cortes J, Cescon DW, Rugo HS, Nowecki Z, Im SA, Yusof MM (2020). Pembrolizumab plus chemotherapy versus placebo plus chemotherapy for previously untreated locally recurrent inoperable or metastatic triple-negative breast cancer (KEYNOTE-355): a randomised, placebo-controlled, double-blind, phase 3 clinical trial. Lancet..

[14] Binnewies M, Roberts EW, Kersten K, Chan V, Fearon DF, Merad M (2018). Understanding the tumor immune microenvironment (TIME) for effective therapy. Nat Med.

[15] Takada K, Kashiwagi S, Goto W, Asano Y, Takahashi K, Hatano T (2018). Significance of re-biopsy for recurrent breast cancer in the immune tumour microenvironment. Br J Cancer.

[16] Hutchinson KE, Yost SE, Chang CW, Johnson RM, Carr AR, McAdam PR (2020). Comprehensive profiling of poor-risk paired primary and recurrent triple-negative breast cancers reveals immune phenotype shifts. Clin Cancer Res..

[17] Szekely B, Bossuyt V, Li X, Wali VB, Patwardhan GA, Frederick C (2018). Immunological differences between primary and metastatic breast cancer. Ann Oncol..

[18] Wang YJ, Fletcher R, Yu J, Zhang L (2018). Immunogenic effects of chemotherapy-induced tumor cell death. Genes Dis..

[19] Murciano-Goroff YR, Warner AB, Wolchok JD (2020). The future of cancer immunotherapy: microenvironment-targeting combinations. Cell Res.

[20] Voorwerk L, Slagter M, Horlings HM, Sikorska K, van de Vijver KK, de Maaker M (2019). Immune induction strategies in metastatic triple-negative breast cancer to enhance the sensitivity to PD-1 blockade: the TONIC trial. Nat Med.

[21] Salgado R, Denkert C, Demaria S, Sirtaine N, Klauschen F, Pruneri G (2016). The evaluation of tumor-infiltrating lymphocytes (TILs) in breast cancer: recommendations by an International TILs Working Group 2014. Ann Oncol.

[22] Park YH, Lal S, Lee JE, Choi YL, Wen J, Ram S (2020). Chemotherapy induces dynamic immune responses in breast cancers that impact treatment outcome. Nat Commun.

[23] Dieci MV, Criscitiello C, Goubar A, Viale G, Conte P, Guarneri V (2014). Prognostic value of tumor-infiltrating lymphocytes on residual disease after primary chemotherapy for triple-negative breast cancer: a retrospective multicenter study. Ann Oncol.

[24] García-Martínez E, Gil GL, Benito AC, González-Billalabeitia E, Conesa MAV, García García TG (2014). Tumor-infiltrating immune cell profiles and their change after neoadjuvant chemotherapy predict response and prognosis of breast cancer. Breast Cancer Res.

[25] Ochi T, Bianchini G, Ando M, Nozaki F, Kobayashi D, Criscitiello C (2019). Predictive and prognostic value of stromal tumour-infiltrating lymphocytes before and after neoadjuvant therapy in triple negative and HER2-positive breast cancer. Eur J Cancer.

[26] Pelekanou V, Carvajal-Hausdorf DE, Altan M, Wasserman B, Carvajal-Hausdorf C, Wimberly H (2017). Effect of neoadjuvant chemotherapy on tumor-infiltrating lymphocytes and PD-L1 expression in breast cancer and its clinical significance. Breast Cancer Res.

[27] Pelekanou V, Barlow WE, Nahleh ZA, Wasserman B, Lo YC, von Wahlde MK (2018). Tumor-infiltrating lymphocytes and PD-L1 expression in pre- and posttreatment breast cancers in the SWOG S0800 Phase II neoadjuvant chemotherapy trial. Mol Cancer Ther.

[28] Schmid P, Rugo HS, Adams S, Schneeweiss A, Barrios CH, Iwata H (2020). Atezolizumab plus nab-paclitaxel as first-line treatment for unresectable, locally advanced or metastatic triple-negative breast cancer (IMpassion130): updated efficacy results from a randomised, double-blind, placebo-controlled, phase 3 trial. Lancet Oncol.

[29] Herrera FG, Ronet C, Ochoa de Olza M, Barras D, Crespo I, Andreatta M (2021). Low-dose radiotherapy reverses tumor immune desertification and resistance to immunotherapy. Cancer Discov.

[30] Zheng W, Skowron KB, Namm JP, Burnette B, Fernandez C, Arina A (2016). Combination of radiotherapy and vaccination overcomes checkpoint blockade resistance. Oncotarget..

[31] Yoneda K, Kuwata T, Kanayama M, Mori M, Kawanami T, Yatera K (2019). Alteration in tumoural PD-L1 expression and stromal CD8-positive tumour-infiltrating lymphocytes after concurrent chemo-radiotherapy for non-small cell lung cancer. Br J Cancer.

[32] Shirasawa M, Yoshida T, Matsumoto Y, Shinno Y, Okuma Y, Goto Y (2020). Impact of chemoradiotherapy on the immune-related tumour microenvironment and efficacy of anti-PD-(L)1 therapy for recurrences after chemoradiotherapy in patients with unresectable locally advanced non-small cell lung cancer. Eur J Cancer.

[33] Ono M, Osako T, Taira S, Shibayama T, Kobayashi K, Kobayashi T (2018). Differences of TILs, hormone receptor, and HER2 status between primary and metastatic tumors. J Clin Oncol.

[34] Liu S, Chen B, Burugu S, Leung S, Gao D, Virk S (2017). Role of cytotoxic tumor-infiltrating lymphocytes in predicting outcomes in metastatic HER2-positive breast cancer: a secondary analysis of a randomized clinical trial. JAMA Oncol.

