# Supplementary material for: Impact of adjuvant chemotherapy and radiotherapy on tumour-infiltrating lymphocytes and PD-L1 expression in metastatic breast cancer
Source: Br J Cancer. 2022 Dec 15;128(4):568–75. doi: 10.1038/s41416-022-02072-2 (PMC9938235; doi:10.1038/s41416-022-02072-2)
Supplement: Supplementary file 1 — All authors agreed with the final manuscript [file 41416_2022_2072_MOESM1_ESM.pdf]

下井先生

よろしくお願いいたします。

Please review the attached final manuscript and choose one of the following

■ I agree with the final manuscript.

☐ I don't agree with the final manuscript.

Your name : Shu Yazaki

Wonderful Tatsunori,

I'm very happy for you and for the team. It's a very informative manuscript, and I of course agree with the final manuscript. Hereunder, more formally.

Please review the attached final manuscript and choose one of the following

- I **agree with the final manuscript.**

Your name : Roberto Salgado

Congrats again!

Roberto

■ I agree with the final manuscript.

☐ I don't agree with the final manuscript.

Your name : Masayuki Yoshida

吉田正行

下井先生

この度は誠におめでとうございます。
I agree with the final manuscript.

大熊ひとみ

下井先生・矢崎先生

おめでとうございます。

■ I agree with the final manuscript.

☐ I don't agree with the final manuscript.

Your name : Yuki Kojima

よろしくお願い致します。

****
小島 勇貴
国立がん研究センター中央病院 腫瘍内科
Yuki Kojima, M.D., PhD.
Department of Medical Oncology, National Cancer Center Hospital, Japan
Tsukiji 5-1-1, Chuo-ku, Tokyo, 104-0045, Japan
TEL: 81-3-3542-2511 (5660), FAX: 81-3-3542-3815
E-mail: yuukojim@ncc.go.jp
****

おめでとうございます。

以下、お願いいたします。

■ I agree with the final manuscript.

☐ I don't agree with the final manuscript.

Your name :　Tadaaki Nishikawa

****
国立がん研究センター中央病院
腫瘍内科　医員
西川忠曉
National Cancer Center Hospital
Department of Medical Oncology
Tadaaki Nishikawa M.D., Ph.D.

〒104-0045　東京都中央区築地 5-1-1
5-1-1 Tsukiji, Chuo-ku, Tokyo, 104-0045, Japan

Tel: +81-3-3542-2511(7846)
Fax: +81-3-3545-3567

E-mail:
tnishika@ncc.go.jp

下井先生、矢﨑先生、皆様

アクセプトおめでとうございます。

■ I agree with the final manuscript.
Your name :　Kazuki Sudo

須藤

下井先生、矢崎先生

アクセプトおめでとうございます。

I agree with the final manuscript.

野口瑛美

下井先生、矢崎先生

おめでとうございます。
共著に加えていただきありがとうございます。

■ I agree with the final manuscript.
□ I don’t agree with the final manuscript.
Your name : Tomoya Kaneda

==
国立がん研究センター中央病院　放射線治療科
金田　朋也
〒104-0045　東京都中央区築地5－1－1
電話：03-3542-2511　Fax：03-3542-3815
E-mail：tomkaned@ncc.go.jp
==

よろしくお願いします。

■ I agree with the final manuscript.

□ I don’t agree with the final manuscript.

Your name:　Sho Shiino

椎野

矢崎先生、下井先生

アクセプトおめでとうございます。
共著に入れて頂きありがとうございます。
下記の記載の仕方でよろしいでしょうか？

The attached paper, which we thank you all for your support, has been accepted by the EJC.
I need your e-mailed agreement on the final version of the paper.

Please review the attached final manuscript and choose one of the following

■ I agree with the final manuscript.

☐ I don't agree with the final manuscript.

Your name :Takeshi Murata

おめでとうございます。

Please review the attached final manuscript and choose one of the following

■ I agree with the final manuscript.

☐ I don't agree with the final manuscript.

Your name : SHIN TAKAYAMA

****
国立研究開発法人
国立がん研究センター中央病院　乳腺外科
医長　高山　伸
〒104-0045　東京都中央区築地 5-1-1
Tel:　03-3542-2511　　ext: 7391
Fax:　03-3542-3815
e-mail: stakayam@ncc.go.jp
****

Please review the attached final manuscript and choose one of the following

■ I agree with the final manuscript.

☐ I don't agree with the final manuscript.

Your name :Yuichiro Ohe

下井先生、矢崎先生

おめでとうございます。

大江

宛先 下井 辰徳

2022/11/10 19:33 にこのメッセージに返信しました。

I agreed it.

Kan Yonemori

The attached paper, which we thank you all for your support, has been accepted by the EJC.
I need your e-mailed agreement on the final version of the paper.

Please review the attached final manuscript and choose one of the following

■ I agree with the final manuscript.

☐ I don't agree with the final manuscript.

Your name : Akihiko Suto

****
国立研究開発法人
国立がん研究センター中央病院
乳腺外科長　首藤昭彦 (すとうあきひこ)
〒104-0045 東京都中央区築地 5-1-1
Tel: 03-3542-2511(代) 内線 7270
Fax:03-3542-3815
E-mail: asuto@ncc.go.jp
