# Supplementary material for: Impact of adjuvant chemotherapy and radiotherapy on tumour-infiltrating lymphocytes and PD-L1 expression in metastatic breast cancer
Source: Br J Cancer. 2022 Dec 15;128(4):568–75. doi: 10.1038/s41416-022-02072-2 (PMC9938235; doi:10.1038/s41416-022-02072-2)
Supplement: Supplementary file 2 — Supplemental material [file 41416_2022_2072_MOESM2_ESM.docx]

**Radiotherapy**

Patients who underwent partial mastectomy were recommended to treat with remaining breast irradiation. For patients who underwent total mastectomy and have ≥T3 or ≥4 axillary lymph node metastasis, chest wall and regional lymph node irradiation (supraclavicular, level III of the axilla) were recommended. If there are 1–3 axillary lymph node metastasis with high-risk features of recurrence based on tumour size, the number of positive lymph nodes, tumour grade and hormone-receptor status, regional lymph node irradiation is also recommended. Although levels I and II of the axilla is not included in the regional lymph node irradiation fields, the level I of axilla is estimated to be covered by the tangential breast and chest wall fields. Treatment was given to the breast area and the regional lymph nodes 5 days a week over 5 weeks, 2.0 Gy per fraction to a total target dose of 50 Gy. If internal mammary lymph node metastasis is clinically suspected, the internal mammary node is also included. For patients with sentinel node metastases but no axillary dissection, we consider including the axilla (e.g., high tangent) in an irradiation field.


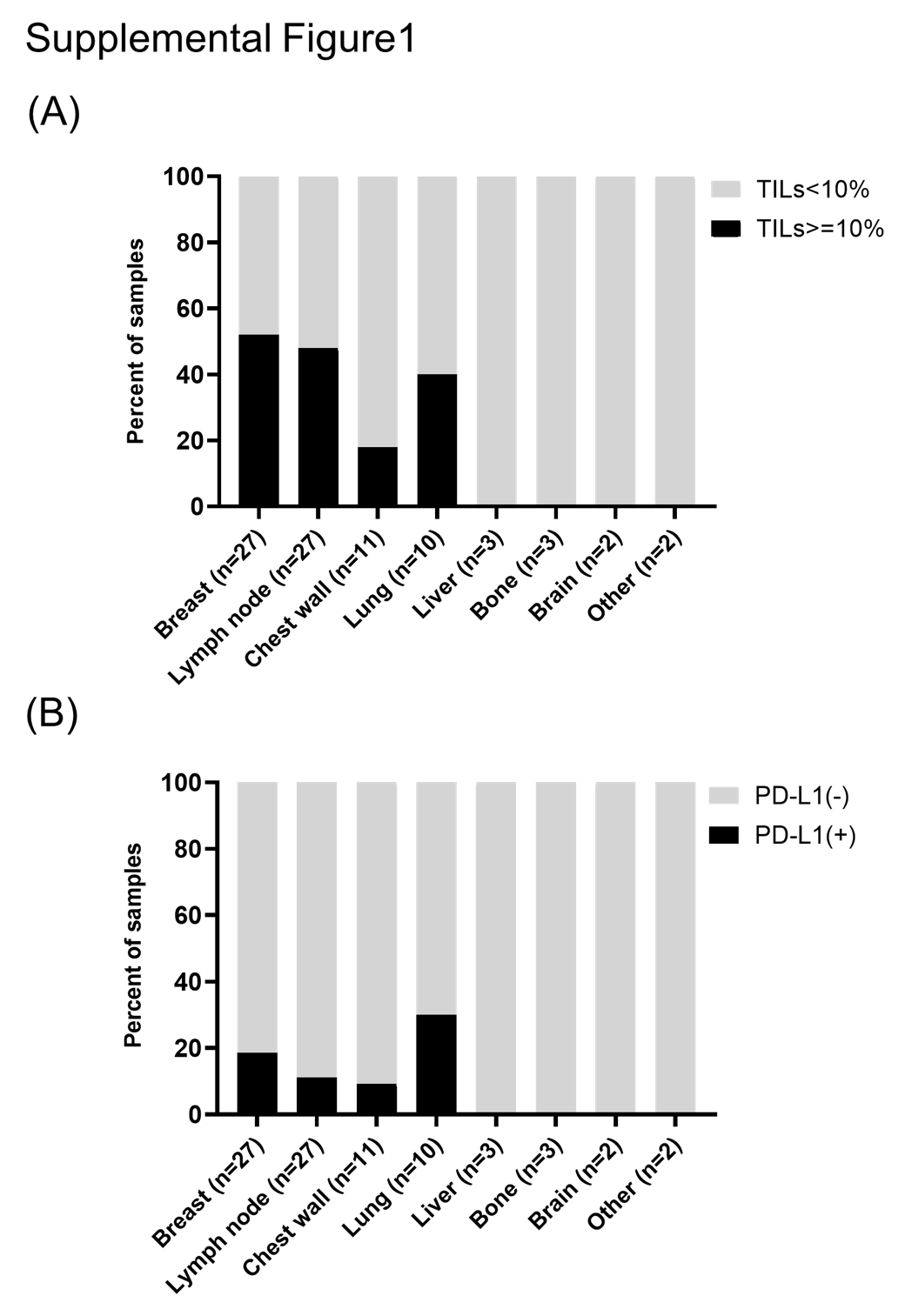


**Supplemental Figure 1. TILs and PD-L1 status by metastatic sites.** Percentage of samples with (A) stromal TILs ≥10% and (B) PD-L1 IC ≥1 by metastatic sites. Abbreviations: IC, immune cell; PD-L1, programmed death-ligand 1; TILs; tumour-infiltrating lymphocytes;


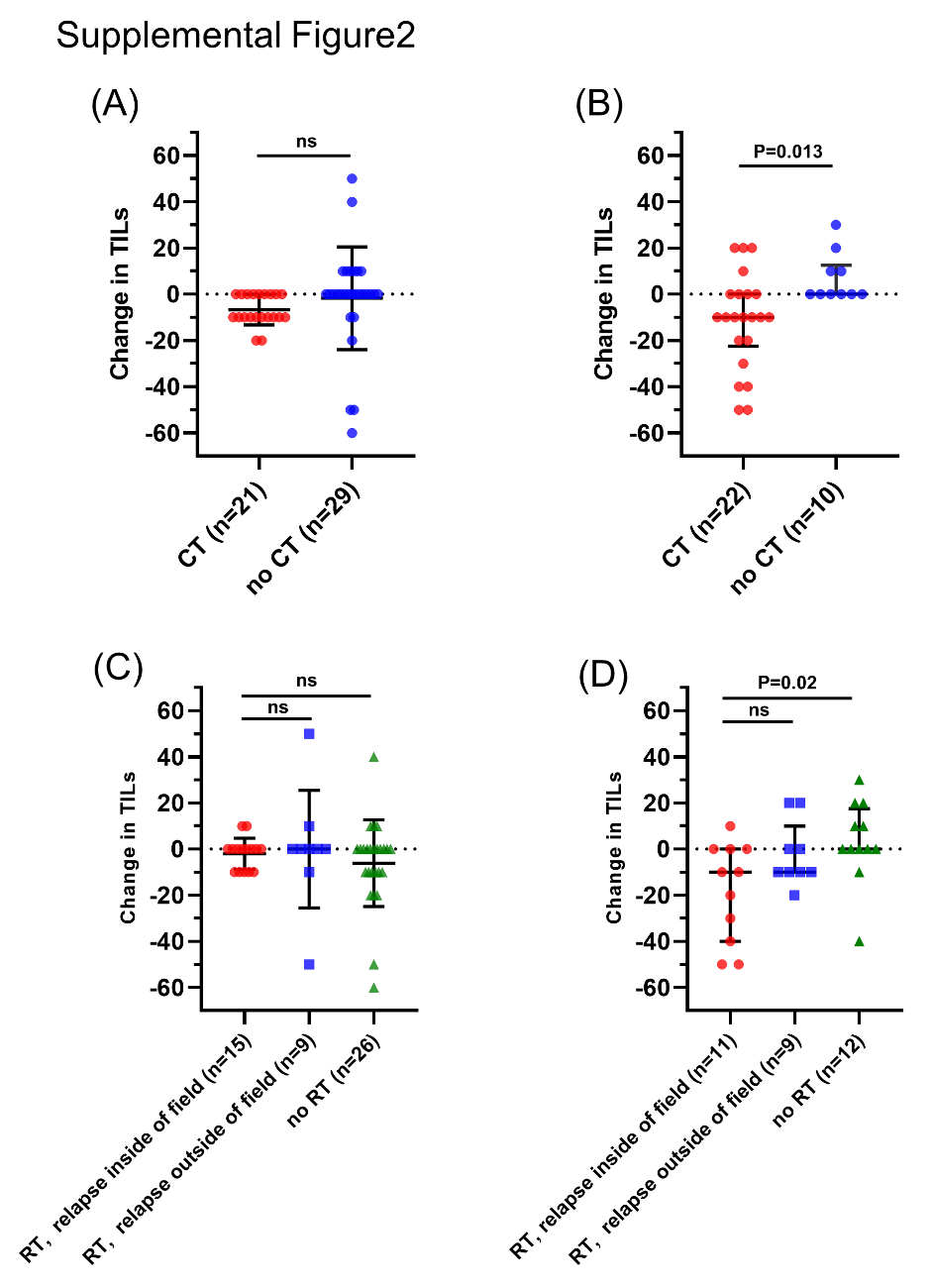


**Supplemental Figure 2. Changes in TILs with or without adjuvant chemotherapy and radiotherapy according to subtype.** Changes in TILs between primary tumours and metastases are compared in patients receiving or not receiving adjuvant chemotherapy in the HR-positive HER2-negative (A) and TNBC (B) groups. Changes in TILs are compared in patients receiving or not receiving adjuvant radiotherapy in the HR-positive HER2-negative (C) and the TNBC (D) groups. The radiotherapy group is divided into two subgroups: inside and outside of the field of recurrence. The bars represent the mean ± SD. Abbreviations: CT, chemotherapy; HR, hormone-receptor; HER2, human epidermal growth factor receptor 2; RT, radiotherapy; SD, standard deviation; TNBC, triple-negative breast cancer; TILs, tumour-infiltrating lymphocytes


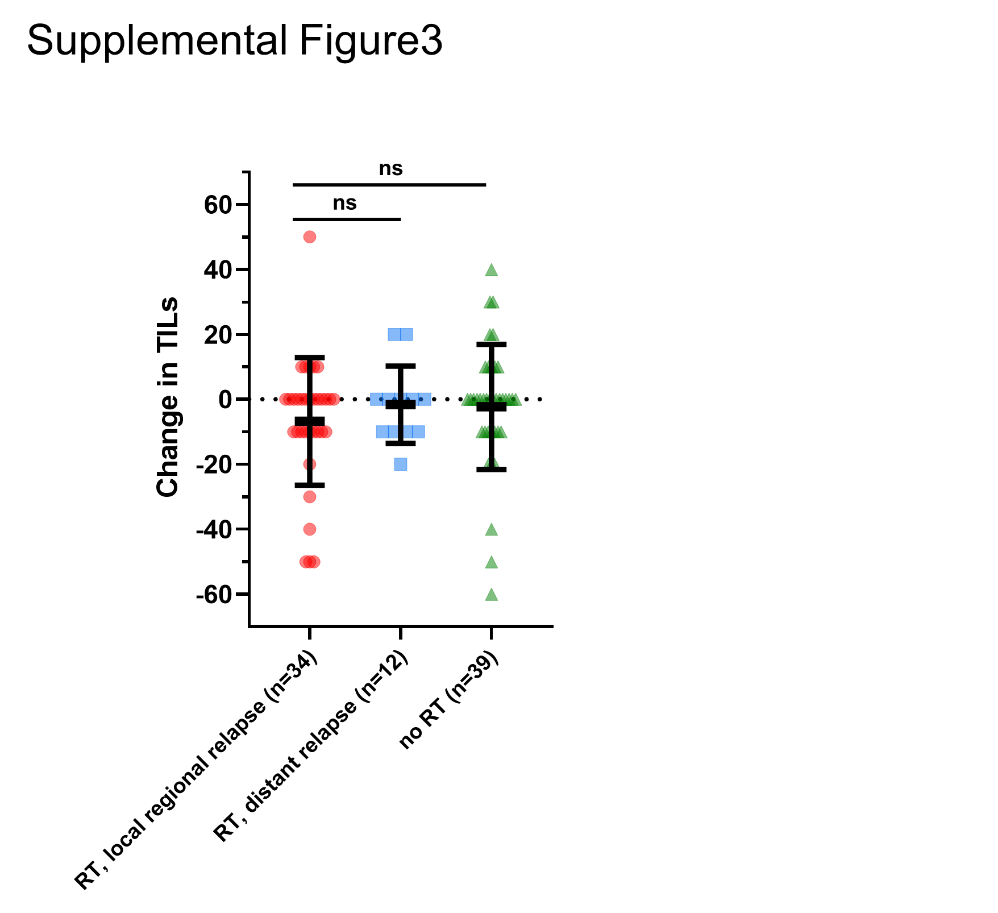


**Supplemental Figure 3. Changes in TILs with or without adjuvant radiotherapy.** Changes in TILs between primary tumours and metastases are compared in patients receiving or not receiving adjuvant radiotherapy. The radiotherapy group is divided into local regional relapse and distant relapse. The bars represent the mean ± SD. Abbreviations: CT, chemotherapy; RT, radiotherapy; SD, standard deviation; TILs, tumour-infiltrating lymphocytes


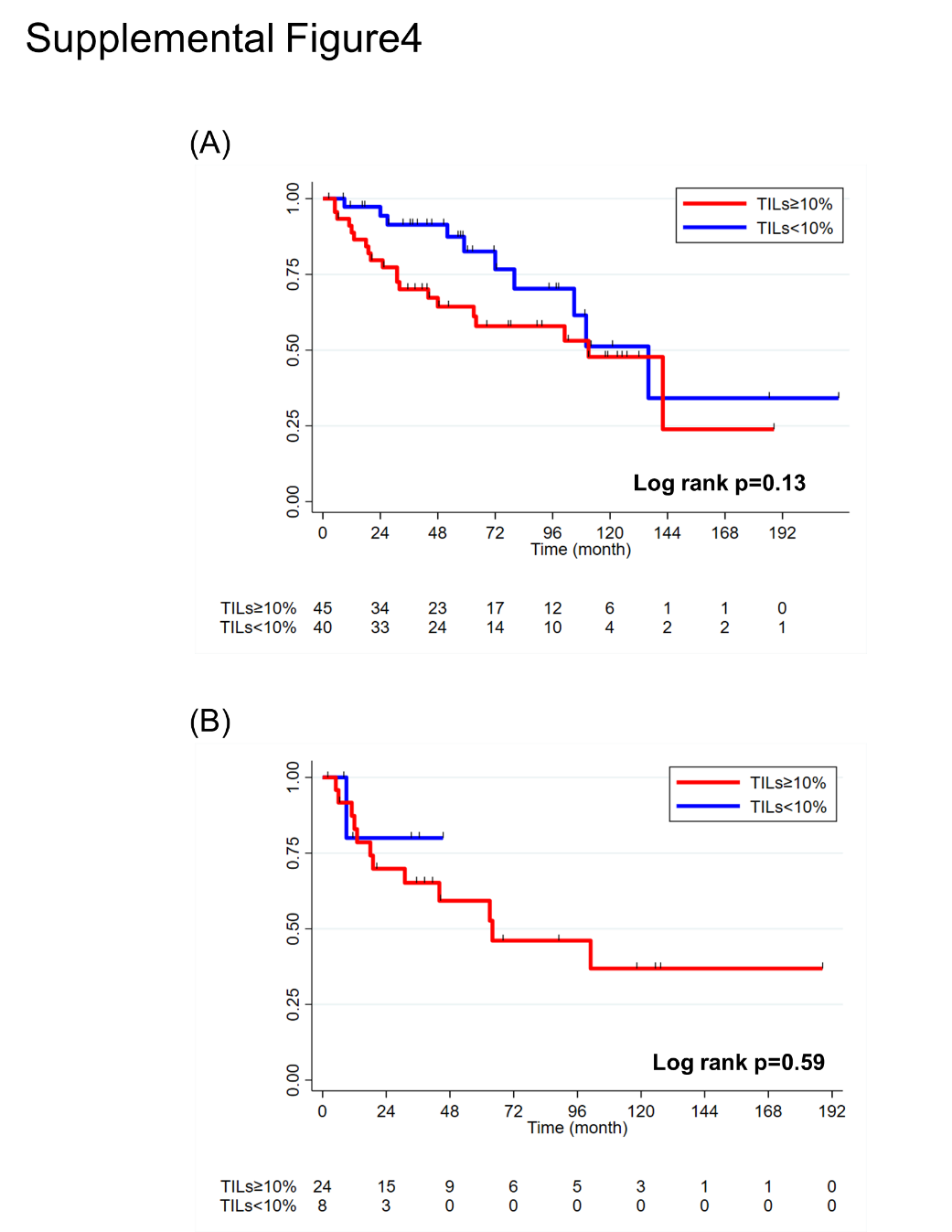


**Supplemental Figure 4. TILs in primary tumours and post-recurrence survival.** Post-recurrence survival in patients with high TILs (≥10%) vs. low TILs (<10%) in primary tumours in the total patient sample (A) and the TNBC subgroup (B). Abbreviations: TILs, tumour-infiltrating lymphocytes; TNBC, triple-negative breast cancer
